# Synergistic Effect of PVDF-Coated PCL-TCP Scaffolds and Pulsed Electromagnetic Field on Osteogenesis

**DOI:** 10.3390/ijms22126438

**Published:** 2021-06-16

**Authors:** Yibing Dong, Luvita Suryani, Xinran Zhou, Padmalosini Muthukumaran, Moumita Rakshit, Fengrui Yang, Feng Wen, Ammar Mansoor Hassanbhai, Kaushik Parida, Daniel T. Simon, Donata Iandolo, Pooi See Lee, Kee Woei Ng, Swee Hin Teoh

**Affiliations:** 1 School of Materials Science and Engineering, Nanyang Technological University, 50 Nanyang Avenue, Singapore 639798, Singapore; yibing001@e.ntu.edu.sg (Y.D.); xinran002@e.ntu.edu.sg (X.Z.); mrakshit@ntu.edu.sg (M.R.); kparida@ntu.edu.sg (K.P.); pslee@ntu.edu.sg (P.S.L.); 2 School of Chemical and Biomedical Engineering, Nanyang Technological University, 62 Nanyang Drive, Singapore 637459, Singapore; luvi0001@e.ntu.edu.sg (L.S.); padmalosini@ntu.edu.sg (P.M.); yang0573@e.ntu.edu.sg (F.Y.); wenfeng@ntu.edu.sg (F.W.); ammar_hassanbhai@osteopore.com (A.M.H.); 3 Laboratory of Organic Electronics, Department of Science and Technology, Linköping University, 601 74 Norrköping, Sweden; daniel.simon@liu.se (D.T.S.); donata.iandolo@emse.fr (D.I.); 4 Mines-Saint-Étienne, Campus Santé Innovations, 10 rue de la Marandière, 42270 Saint-Priest-en-Jarez, France; 5 Center for Nanotechnology and Nanotoxicology, Harvard T.H. Chan School of Public Health, Harvard University, 677 Huntington Avenue, Boston, MA 02115, USA; 6 Environmental Chemistry and Materials Centre, Nanyang Environment and Water Research Institute, Nanyang Technological University, 1 Cleantech Loop, CleanTech One, Singapore 637141, Singapore; 7 Lee Kong Chian School of Medicine, Nanyang Technological University, 59 Nanyang Drive, Singapore 636921, Singapore

**Keywords:** electrical stimulation, pulsed electromagnetic field, piezoelectric scaffold, bone tissue engineering, electroactive biomaterial, stimuli-responsive materials

## Abstract

Bone exhibits piezoelectric properties. Thus, electrical stimulations such as pulsed electromagnetic fields (PEMFs) and stimuli-responsive piezoelectric properties of scaffolds have been investigated separately to evaluate their efficacy in supporting osteogenesis. However, current understanding of cells responding under the combined influence of PEMF and piezoelectric properties in scaffolds is still lacking. Therefore, in this study, we fabricated piezoelectric scaffolds by functionalization of polycaprolactone-tricalcium phosphate (PCL-TCP) films with a polyvinylidene fluoride (PVDF) coating that is self-polarized by a modified breath-figure technique. The osteoinductive properties of these PVDF-coated PCL-TCP films on MC3T3-E1 cells were studied under the stimulation of PEMF. Piezoelectric and ferroelectric characterization demonstrated that scaffolds with piezoelectric coefficient *d*_33_ = −1.2 pC/N were obtained at a powder dissolution temperature of 100 °C and coating relative humidity (RH) of 56%. DNA quantification showed that cell proliferation was significantly enhanced by PEMF as low as 0.6 mT and 50 Hz. Hydroxyapatite staining showed that cell mineralization was significantly enhanced by incorporation of PVDF coating. Gene expression study showed that the combination of PEMF and PVDF coating promoted late osteogenic gene expression marker most significantly. Collectively, our results suggest that the synergistic effects of PEMF and piezoelectric scaffolds on osteogenesis provide a promising alternative strategy for electrically augmented osteoinduction. The piezoelectric response of PVDF by PEMF, which could provide mechanical strain, is particularly interesting as it could deliver local mechanical stimulation to osteogenic cells using PEMF.

## 1. Introduction

Bone is the second most transplanted organ worldwide. The global bone substitute market was valued at USD 2.9 billion in 2019 and is expected to expand at 3% annually until 2030. This sector represents a huge personal and economic burden on the healthcare sector owing to aging societies and an increasing prevalence of obesity and osteoporosis. Therefore, bone lesion treatments that are effective, biocompatible, and osteogenic are in great demand [1]. Among all engineered bone grafts, polycaprolactone (PCL)-based scaffolds are well established to host osteogenic and chondrogenic cell growth, proliferation, and differentiation [2,3]. PCL-based scaffolds have been used in a number of successful clinical applications, such as cranioplasty and long bones’ regeneration [4,5]. PCL materials have rheological properties that allow them to be produced in various forms such as films, nanofibers, and macroporous 3D-printed scaffolds, being versatile in a variety of culture conditions, including small-scale culture plates and lab-scale bioreactors [6,7]. Bioactive ceramics such as β-tricalcium phosphate (TCP) can be incorporated into PCL for better osteogenesis and improved mechanical properties [8]. The release of calcium and phosphate ions grants the scaffolds cell chemotaxis, recruiting cells to grow in the implanted site [9].

Smart or stimuli-responsive materials have emerged as exciting possibilities in various biomedical applications such as drug delivery [10], biosensors [11], and tissue engineering [12]. They provide versatility in terms of tuneable properties such as mechanical, structural, electrical, and biochemical responses to internal or external stimuli. As bone possesses piezoelectricity [13], biomimetic scaffolds fabricated using piezoelectric materials that could respond to electrical or mechanical stimuli are of great interest for bone tissue engineering. In piezoelectric materials, surface charges are altered upon application of mechanical deformation owing to dipole centre separation. When these materials are implanted, these surface charges provide electrical cues locally to the damage sites, promote protein adhesion, modify membrane potential, regulate the voltage-gated calcium channels, and subsequently modulate cell behaviours [14]. They are shown to effectively control cell adhesion to the substrate, enhance cell proliferation, and trigger osteoinduction and bone regeneration in various studies [14,15,16,17]. Polyvinylidene difluoride (PVDF) is a promising candidate owing to its high piezoelectricity, thermal stability, and biocompatibility. PVDF can crystalized to three main polymorphs phases: trans and gauche (TG^+^TG^−^) for α, all-trans (TTTT) for β, and (T_3_G^+^T_3_G^−^) for γ, among which β phase is the most electroactive phase and could be further polarized to align the electric dipoles and generate piezoelectric response [18]. Current polarization strategies of piezoelectric PVDF include mechanical stretching, annealing, high electric field inducing, and electrospinning [18]. These techniques require high temperature or high voltage, which restricts PVDF to be used in combination with other clinically successful bone grafts, such as microporous PCL-TCP scaffolds with low melting temperature, and calls for the development of more versatile methods.

In some clinical cases, patients are restricted in movements owing to serious health conditions, such that natural mechanical stimulation is lacking and the effects of piezoelectric scaffolds are reduced. Such limitation calls for additional measures of remote mechanical or electrical stimulation of cells and tissues [19]. Electromagnetic fields (EMFs), especially pulsed electromagnetic fields (PEMFs), not only provide such a tool to electrically stimulate piezoelectric scaffolds to deliver local mechanical stimulation, but also offer additional advantages of remote electrical stimulation of cell proliferation and cell differentiation without changing local pH or creating reactive oxygen species [20]. Although the underlying mechanism has not been fully understood, a series of preclinical and clinical experiments have shown that PEMF at specific intensities and durations, as a single stimulus, was able to influence cellular response by affecting membrane electrical potential and activating various signaling pathways, increasing cell proliferation [21], up-regulating osteogenic gene expression [22], and promoting bone formation [23]. Thus, PEMF has the potential to enhance cellular responses to biomaterial scaffolds or bone grafts by synergistically positively affecting cell growth and differentiation.

However, current understanding of cellular response under the combined influence of PEMF and piezoelectric properties in scaffolds is still lacking. In-depth cell analysis, including osteogenic differentiation and gene expression in a culture period beyond 14 days, is limited in current studies [14,24]. Hence, in the present study, we aimed to gain insight into the cell–material interaction and osteogenic potential of piezoelectric scaffolds coupled with PEMF on MC3T3-E1 murine pre-osteoblasts cell culture and stimulation. Specifically, PCL-TCP coating with a piezoelectric PVDF conformal film was optimized to guarantee its efficacy to provide electrical stimulations while retaining the advantages of PCL-TCP scaffolds. PEMF was generated by solenoid coils and exposed horizontally to the cells loaded on piezoelectric scaffolds.

## 2. Results

### 2.1. Generation of β-PVDF by Polar Solvent

**Figure 1:**
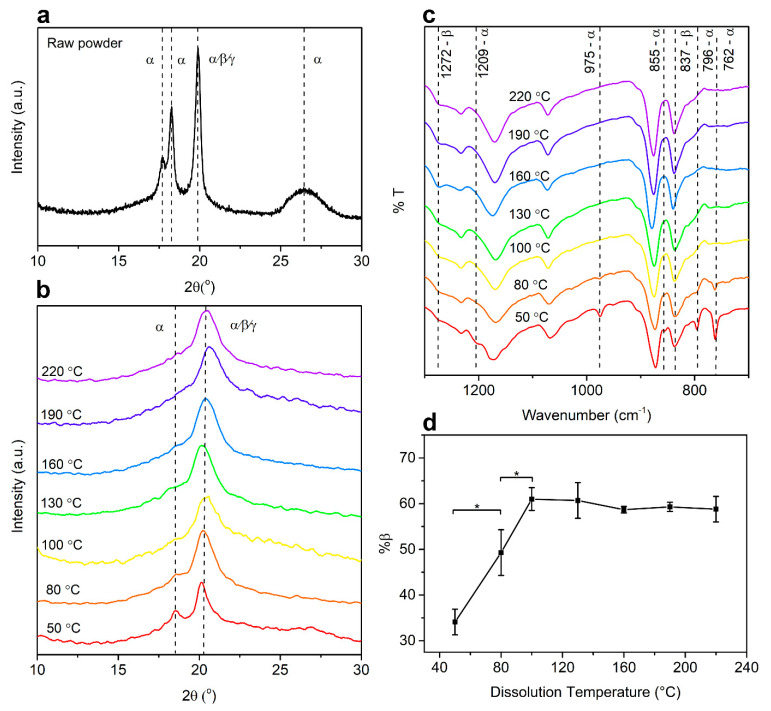
Phase characterization of polyvinylidene fluoride (PVDF). (**a**) X-ray diffraction (XRD) spectra indicating the characteristic peaks of α-PVDF and β-PVDF for raw PVDF powder. (**b**) XRD spectra and (**c**) Fourier-transform infrared spectroscopy (FTIR) spectra of PVDF films fabricated at dissolution temperatures of 50 to 200 °C. (**d**) Comparison of β-PVDF content at increasing dissolution temperatures (* *p* < 0.05, *n* = 3).

The characterizations were done on the air-dried films prepared by solvent-casting of PVDF solution in dimethyl sulfoxide (DMSO) dissolved at various temperatures. X-ray diffraction (XRD) analysis is often used to determine the phases of PVDF, which normally show intense peaks around 2θ = 20° corresponding to the diffraction plane of (110/200). The characteristic peak for the β phase usually appears at 20.3°, while the α phase shows not only the peak at 19.9°, but also additional peaks around 17.7°, 18.3°, and 26.6° corresponding to the diffraction planes of (100), (020), and (021), respectively. The γ phase has been reported to present similar peaks as the α phase at 18.5°, 19.2°, 20.0°, and 26.8°, associated with the planes (020), (200), (110), and (022), respectively [25]. It was observed from Figure 1a and Supplementary Table S1 that, in the raw PVDF powder, all characteristic peaks corresponding to the α phase were presented, confirming the α phase as the dominant phase in the raw PVDF powder. The ferroelectric β phase is unstable, thus PVDF favorably crystallizes into a non-ferroelectric α phase. After dissolution in DMSO at 50 °C, as shown in Figure 1b, the intensity of the peaks at 26.2° decreased and the first peak shifted to 18.5°, which is the characteristic peak of the γ phase, indicating the possible co-existence of both phases in this state. At a dissolution temperature of 80 °C, the peak at 18.60° presented as a shoulder and the peak around 26.6° disappeared. From a dissolution temperature of 100 °C and above, a dominant peak around 20.3° was present [26].

In addition to XRD analysis, Fourier-transform infrared spectroscopy (FTIR) and differential scanning calorimetry (DSC) were conducted to quantify the fraction of β phase and crystallinity of PVDF. It could be observed from Figure 1c and Supplementary Table S2 that, with increasing dissolution temperature, a higher total amount of β phase was generated up to 100 °C as a result of improved crystallinity and fraction of β phase in all phases. In the FTIR spectra shown in Figure 1c, vibrational bands corresponding to the α phase were visible at 762 cm^−1^, 796 cm^−1^, 855 cm^−1^, 975 cm^−1^, and 1209 cm^−1^, while vibrational bands corresponding to the β phase were observed at 837 cm^−1^ and 1272 cm^−1^ [26]. From the graph, the characteristic peaks for the α phase disappeared starting from 100 °C, with an accompanying slight increase of β phase characteristic peaks, indicating the formation of β phase with increasing temperature. The melting temperatures of the α and β phases have been reported to be similar at 167–172 °C, while the γ phase was reported to have a melting temperature of 179–180 °C. Therefore, DSC was not used to distinguish different phases, but rather the overall crystallinity [25,26]. By calculating the crystallization enthalpy and crystallinity from DSC results, an increasing trend was observed with increasing temperature, as shown in Supplementary Table S2. The relative quantification of the β phase (%β) in Figure 1d further confirmed this transformation as the %β phase expanded with increasing temperature up to 100 °C.

### 2.2. Surface Properties

**Figure 2:**
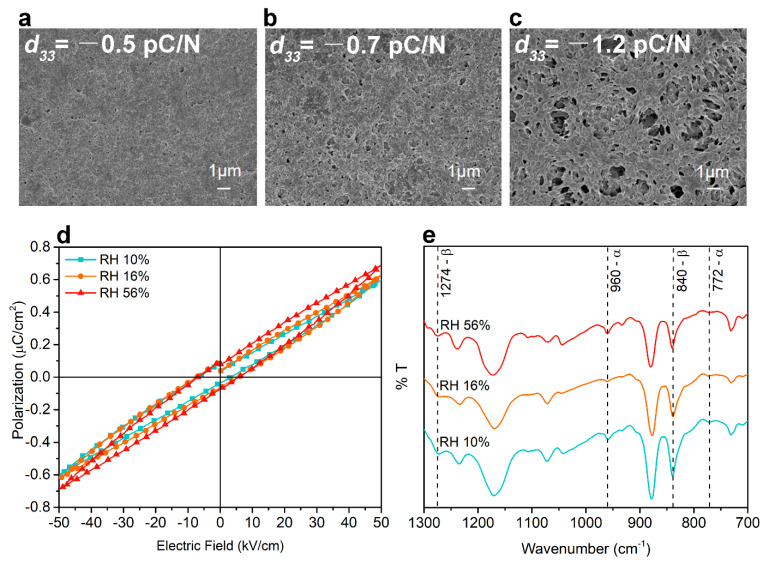
Surface morphology characterized by scanning electron microscopy (SEM) of PVDF-coated polycaprolactone (PCL)-tricalcium phosphate (TCP) films fabricated at a relative humidity (RH) of (**a**) 10%, (**b**) 16%, and (**c**) 56%. (**d**) Representative ferroelectric polarization–electric field (P–E) hysteresis measured under an applied field up to 50 kV/cm. (**e**) FTIR spectra of PVDF-coated PCL-TCP films.

Representative field emission scanning electron microscopy (FESEM) images of samples conditioned at three relative humidity (RH) levels (10%, 16%, and 56%) are shown in Figure 2a–c. It can be observed that, in the RH 10% group, a relatively homogeneous film was obtained with the presence of a few small-sized pores. With the increasing humidity level, a more porous structure presented in the surface and loosely connected PVDF nodules appeared. This is supported by surface topography characterization by atomic force microscopy (AFM), shown in Supplementary Figure S2. Wettability of PVDF coated PCL-TCP films (Supplementary Table S5) was measured and they were shown to be hydrophobic, as reported previously [27]. The water contact angles (WCAs) of all three groups were larger than pristine PCL-TCP films (91.4° ± 4.1°). There was no significant difference between WCA of the three sample groups. However, a slight decreasing was evident, which might be attributed to increased pore size and polarization of the film owing to the increased surface charge in the more poled samples [28].

### 2.3. Ferroelectric and Piezoelectric Behavior

The macroscopic ferroelectric hysteresis measurements were conducted on PVDF-coated PCL-TCP films conditioned at the three RH levels (10%, 16%, and 56%). The presence of a ferroelectric P–E loop in Figure 2d indicated the presence of the ferroelectric domain in the samples following their coating with the modified breath figure method. With increasing humidity, the maximum polarization (Pmax) and remnant polarization (Pr) also increased, as shown in Table S3. A maximum Pr of 0.09 μC/cm^2^ at an electric field of 50 kV/cm was present in the RH 56% group, being two orders of magnitude smaller than that reported for β-phase PVDF thin film, having a Pr of 6.95–8.41 μC/cm^2^ at an electric field of 4000 kV/cm [29,30]. The coercive fields (Ec) of samples at all three conditions were similar to the reported Ec value for spin-coated PVDF thin films from 50 to 115 kV/mm [30,31,32]. Besides, samples at all three conditions showed relaxor ferroelectric properties based on the lack of a discernible squarish ferroelectric loop, indicating a lack of longer-range order in the ferroelectric domain. The values obtained for the piezoelectric *d*_33_ coefficient for RH 10%, 16%, and 56% samples were −0.5 ± 0.1 pC/N, −0.7 ± 0.1 pC/N, and −1.2 ± 0.3 pC/N, respectively. As the calculated fraction of β phase was not significantly different among the three sample groups, as shown in Supplementary Table S4, a self-polarization effect was likely occurring owing to the modified breath figure method [33].

### 2.4. Cell Morphology

**Figure 3:**
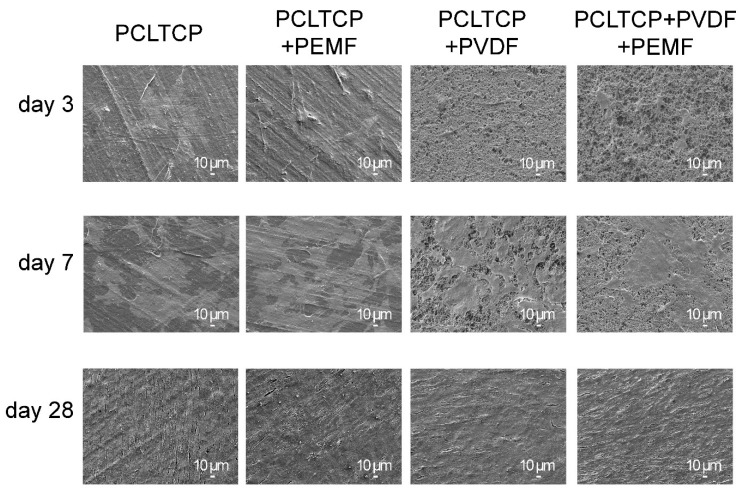
Fixed cell morphology on PCL-TCP without (PCL-TCP) and with pulsed electromagnetic field (PEMF) (PCL-TCP+PEMF) and on PVDF-coated PCL-TCP without (PCL-TCP+PVDF) and with PEMF (PCL-TCP+PVDF+PEMF) observed using SEM on day 3, 7, and 28.

Scaffolds coated at a dissolution temperature of 100 °C and RH of 56% were used for cell culture characterizations owing to the highest piezoelectricity obtained. The interaction between cells and substrates was analyzed using FESEM on day 3, 7, and 28, as shown in Figure 3. It was observed that the cells adhered and spread on the surface of both substrates and displayed characteristic star/slayed-shaped morphologies, indicating biocompatibility of the coated films with and without PEMF exposure. On day 7, the cells reached around 80% confluency and intercellular connections were observed for all groups. On day 28, the cells showed spindle-shaped morphology and packed as cell density increased and formed confluent monolayer sheets for all groups.

### 2.5. Cell Metabolic Activity

**Figure 4:**
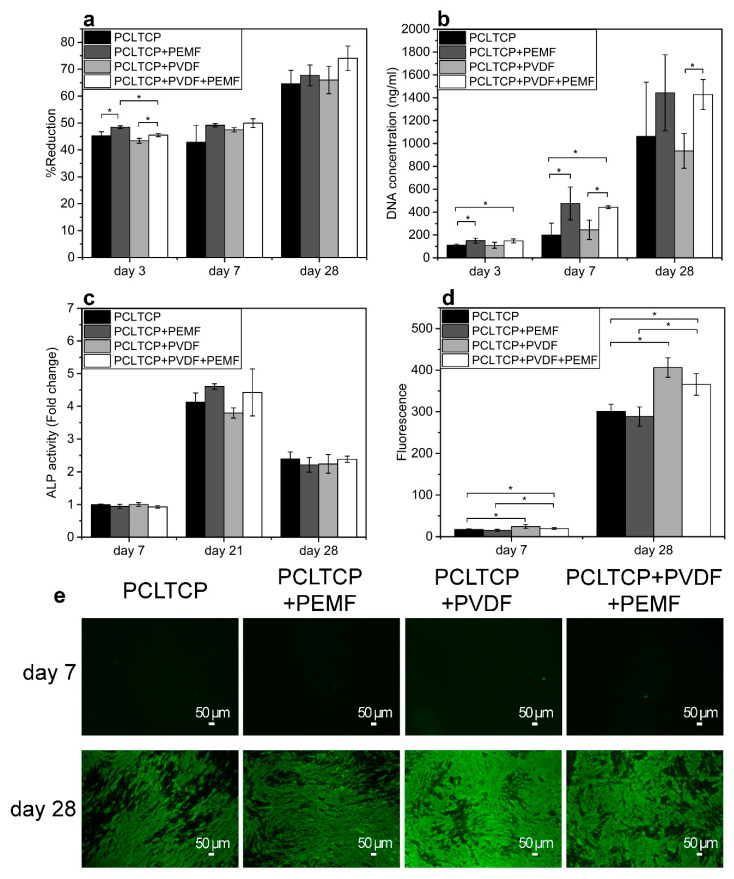
(**a**) Cell metabolic activity by measuring the percentage of alarmaBlue reduction. (**b**) Cell proliferation quantified by DNA quantification. (**c**) Alkaline phosphatase (ALP) activity. (**d**) Quantification of Osteoimage fluorescence. (**e**) Osteoimage staining of hydroxyapatite rich regions (green). Sample groups: PCL-TCP without (PCL-TCP) and with PEMF (PCL-TCP+PEMF), PVDF-coated PCL-TCP without (PCL-TCP+PVDF) and with PEMF (PCL-TCP+PVDF+PEMF) (* *p* < 0.05, *n* = 3).

The alamarBlue reduction results in Figure 4a indicate that the metabolic activity of cells increased with PEMF exposure and slightly decreased with PVDF incorporation on day 3. On day 7 and 28, although no significant difference was observed, electrically stimulated groups exhibited higher levels of cell metabolic activities, with the PCL-TCP+PVDF+PEMF group showing the highest.

### 2.6. Cell Proliferation

Cell proliferation was characterized by DNA quantification (Figure 4b). The groups exposed to PEMF showed significantly enhanced cell proliferation on both substrates at all time points, as indicated by higher DNA content. PVDF on its own did not exert a significant impact on cell proliferation.

### 2.7. Cell Differentiation and Mineralization

Alkaline phosphatase (ALP) activity is an early osteoblastic differentiation marker that plays an essential role in the cleavage of organic phosphate esters and the formation of mineralized nodules [34]. An additional time point on day 21 was added as ALP activity of MC3T3-E1 cells has been reported to peak on day 21 [35]. On day 7 and 28, all groups exhibited similar ALP activities (Figure 4c). However, it could be observed that, on day 21, ALP activity tended to be higher in the PCL-TCP+PEMF group compared with the PCL-TCP group, although the difference was not statistically significant. Likewise, the PCL-TCP+PVDF+PEMF group appeared to register higher ALP activity than the PCL-TCP+PVDF group. Similar to earlier observations, PVDF on its own did not affect ALP activity.

Cell mineralization was characterized by the formation of hydroxyapatite nodules in the extracellular matrix (ECM), which could be visualised and quantified by OsteoImage assay, which selectively stains hydroxyapatite. Fluorescence intensities on day 7 and 28 were quantified at the early and late stages of mineralization (Figure 4d,e). It can be observed that, on day 28, prominent bright green areas indicated wide-spread mineralization across the material, whereas on day 7, limited green spots indicated the early formation of hydroxyapatite nodules, which was initiated only in localized cell confluent regions of the scaffold. Quantification of fluorescence intensity showed that, when compared with PCL-TCP, PCL-TCP+PVDF induced more hydroxyapatite deposition regardless of PEMF exposure and time points, while PEMF alone did not exert a significant effect on mineralization.

### 2.8. Osteogenic Gene Expression

**Figure 5:**
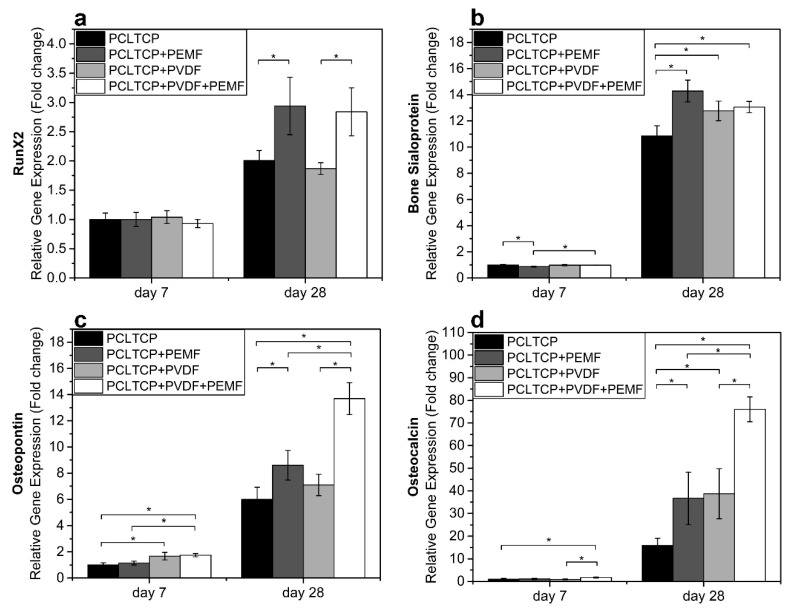
Osteogenic gene expression of early differentiation markers (**a**) *RUNX2* and (**b**) bone sialoprotein (*BSP*) and late bone differentiation markers (**c**) osteocalcin (*OC*) and (**d**) osteopontin (*OP*). Sample groups: PCL-TCP without (PCL-TCP) and with PEMF (PCL-TCP+PEMF), PVDF-coated PCL-TCP without (PCL-TCP+PVDF) and with PEMF (PCL-TCP+PVDF+PEMF) (* *p* < 0.05, *n* = 3).

Transcriptional levels of four osteogenic differentiation-related genetic markers were evaluated and quantified in our experiment, namely, runt-related transcription factor 2 (*RUNX2*), bone sialoprotein (*BSP*), osteopontin (*OP*), and osteocalcin (*OC*). Overall, significant upregulations of all gene markers were observed from day 7 to day 28, as shown in Figure 5. From the results, PEMF induced significantly higher *RUNX2* expression on both PCL-TCP and PCL-TCP+PVDF groups on day 28 (1.5-fold change), while PVDF alone did not exert any effects on the expression of this gene. It could be observed that, on day 7, all stimulated groups showed slightly lower *BSP* expression, while on day 28, at the peak of mineralization, both PEMF and PVDF stimulation induced higher *BSP* expression. The effect of the stimulation was more remarkable for *OC* and *OP*, the late osteogenic gene markers. Either PEMF or PVDF, as a single stimulus, was able to induce higher expression of these markers, yet the synergistic effect of PEMF and PVDF enhanced the upregulation significantly.

The effect of PEMF and PVDF coating on osteogenesis is summarized in Table 1. Compared with the control group of pristine PCL-TCP scaffold, PEMF, as a single stimulus, positively impacts cell metabolic activity, cell proliferation, and osteogenic gene expression, whereas PVDF coating, as a single stimulus, mainly enhances cell mineralization and osteogenic gene expression. The combination of both stimuli leads to better performance than using any single stimuli, with the proliferation and expression of late osteogenic gene OC and OP most significantly boosted. The results in our study provide evidence that PEMF and PVDF could synergistically enhance osteogenesis of MC3T3-E1 cells in various aspects, including metabolic activity, proliferation, mineralization, and osteogenic gene expression, potentially leading to more efficacious bone regeneration.

## 3. Discussion

**Figure 6:**
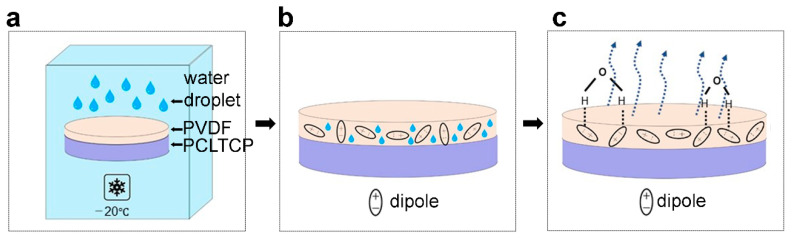
Schematic of the self-poling effect of PVDF-coated PCL-TCP scaffold fabricated using modified breath-figure method. (**a**) Scaffolds are conditioned in a closed chamber at −20 °C. (**b**) Water droplets sink into the polymer matrix and interacted with PVDF molecular dipoles. (**c**) Water molecules evaporate and leave the polymer surface, lifting negative dipoles by hydrogen bonding.

Piezoelectric PVDF-coated PCL-TCP scaffolds were prepared by a modified breath-figure technique at low temperatures, where dissolving temperature and relative humidity impacted the level of piezoelectric properties. FTIR, DSC, and XRD results demonstrated a dissolution temperature of 100 °C as being optimal in obtaining the greatest amount of β phase in the PVDF films. Ferroelectric and piezoelectric characterization revealed that a high relative humidity of RH 56% was preferred to obtain self-poled scaffolds with the piezoelectric coefficient *d*_33_ of −1.2 pC/N. Porous morphology and rough surfaces were observed by both SEM and AFM characterizations in all the prepared scaffolds. In the PVDF/solvent system, polar solvents such as DMSO dissolved PVDF via dipole interaction with CH_2_CF_2_ or hydrogen bonding. They can disrupt the van der Waal’s forces that hold the dipoles together, thus creating room for chain movements. Furthermore, this interaction can also reduce the energy barrier for the formation of all trans- conformation β-PVDF, and thus rotates the strong C–F bonds [36]. This interaction has been reported to be enhanced at elevated temperatures or higher solvent concentration and polarity, which contributed to increasing interactions between PVDF and solvent [37,38]. It could be interpreted from our experimental results that, at temperatures below 100 °C, the temperature was the limiting factor for PVDF–DMSO interaction. Therefore, by increasing the temperature, the PVDF/solvent interactions were enhanced and more β phase content was generated. At temperatures above 100 °C, the solvent concentration became the limiting factor. As DMSO concentration was kept constant for all dissolution temperatures, the amounts of β phase generated did not change as well. The breath-figure method was conducted at a low temperature, which facilitated water-induced polarization without application of a high electric field or high temperature. The proposed mechanism is demonstrated in Figure 6. When PVDF/DMSO solution-coated films are conditioned inside the closed chamber at −20 °C (Figure 6a), water vapors condense onto the solution and penetrate among the polymer chains, acting as a template for the polymer to nucleate around (Figure 6b). Therefore, with a higher water vapor content, more water sinks into the polymer chains and induces voids when water molecules escape the polymer surface during the drying step. This can be indicated by increased pore density on the material surface, demonstrated by SEM and AFM. The self-polarizing behavior could be attributed to the interaction between molecular dipoles in PVDF and condensed water molecules. In PVDF, a pair of electronegative fluorines and electropositive hydrogens attached to the carbon backbone acts as the molecular dipoles. When water molecules condense onto the polymer, hydrogen bonds are formed between the O-H group and electronegative fluorines in PVDF [33]. When water molecules escape the polymer surface during evaporation (Figure 6c), they lift the electronegative fluorines upwards and trigger the alignment of PVDF dipoles. Hydrogen bonding has been widely applied to elicit the self-alignment of PVDF dipoles in Langmuir–Blodgett deposited PVDF [39], PVDF-Cerium composite film [40], PVDF-AlO-rGO nanocomposite [41], and PVDF-TrFE sponge [33]. Films prepared by the breath-figure method exhibited a relatively low piezoelectric coefficient compared with PVDF films obtained using other methods such as corona poling or annealing [42,43], but its magnitude is comparable to that of natural bone (0.7 pC/N) [44]. Compared with other poling methods such as corona poling, annealing, and cold drawing, the modified breath-figure method facilitates PVDF to be poled on surfaces of complex structures and at low temperatures, which is versatile for use in various applications including three-dimensional structure or low melting temperature materials.

**Figure 7:**
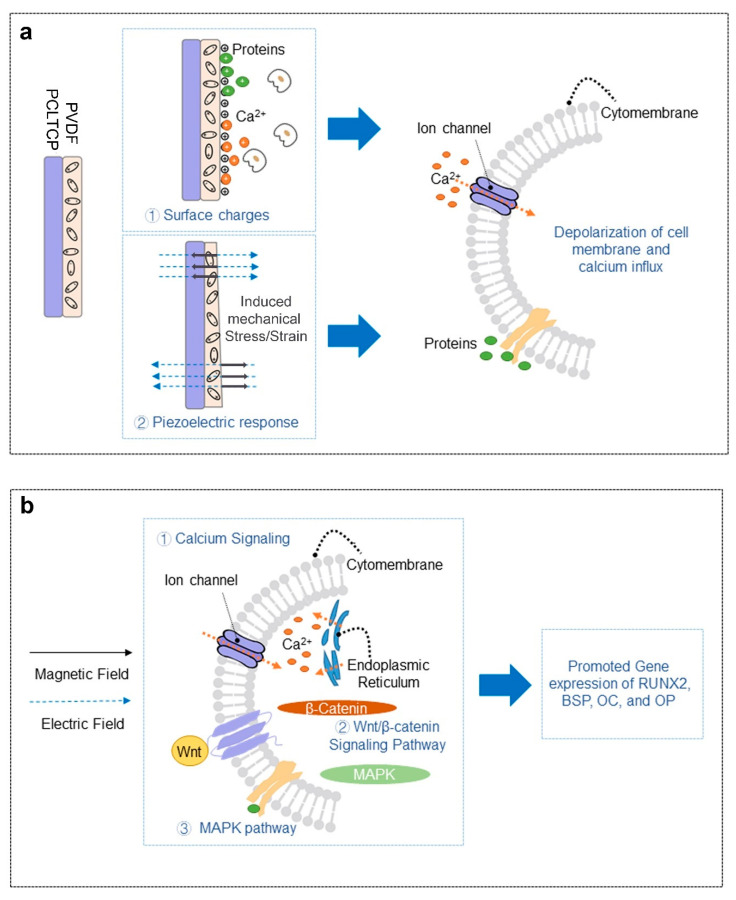
Schematic of the effects of (**a**) PVDF-coated PCL-TCP and (**b**) PEMF on cells.

The developed piezoelectric scaffolds were then evaluated under PEMF exposure for MC3T3-E1 pre-osteoblast osteogenesis. The results demonstrated that PVDF-coated groups possessed good cell adhesion and healthy cell morphology (spindle-shaped), as can be observed on SEM images (Figure 3), and led to the conclusion of a good biocompatibility. A generally improved osteogenesis was observed for PVDF-coated groups as compared with the PCL-TCP control groups, as determined from hydroxyapatite staining by OsteoImage and osteogenic gene expressions. We hypothesize that the positive cellular response could be attributed to the depolarization of cell membrane resulting from two factors: the surface charges and PVDF piezoelectric response to the applied electric field that generates mechanical stress/strain on the surfaces. This is schematically illustrated in Figure 7a. It has been reported that both surface charges and mechanical stimulations are able to alter electric charge distribution across cell membranes, which is commonly regarded as cell membrane depolarization, and lead to the opening of calcium channels with consequent calcium influx [45]. Herein, we first proposed that the distribution of negative charges on the surface has resulted from aligned dipoles in β-PVDF. Cations including calcium and positively charged proteins or peptides could adhere to the negatively charged surface as a result of electrostatic interaction. Previous studies on cell–PVDF interactions have demonstrated the ability of PVDF alone to influence cellular activities. Zhou et al. revealed the beneficial effects of PVDF surface charges on the expression of osteogenic genes [43]. Various studies reported that the formation of the electroactive phase of PVDF effectively induced mineralization [16,43,46]. Secondly, with the external electric fields passing through the PVDF layer in the direction of the aligned dipoles, mechanical strains will be induced and impacted on the cell attachment and proliferation. The induced strain is estimated to be 1.7 to 2.3 × 10^−9^ ppm (detailed calculation in Supplementary Information). This induced strain of PVDF by PEMF provided local mechanical stimulations to osteogenic cells. In cases where patients lack the natural mechano-electrical stimulations from walking, jumping, and running, the implantation of piezoelectric implants such as PVDF-coated scaffolds may enable the tissue to be stimulated mechanically from outside of the human body using PEMF. The magnetic field-induced strain might take part in the gene upregulation observed in our study, which is first time reported that even a low magnitude of strain might have influenced osteogenesis at the cellular level.

In our study, we observed that PEMF exposure exerted a synergistic effect when cells seeded on piezoelectric scaffolds were treated. Over the past decades, the impact of PEMF alone on osteogenesis has been studied for different cell types with a wide range of parameters, with both suppressive and enhancement effects reported. Jansen et al. performed PEMF (15 Hz) exposure on human bone marrow-derived stromal cells and found PEMF could enhance mineralization at the expense of proliferation [47]. In contrast, when Ferroni et al. applied PEMF using a miniaturized electromagnetic device on mesenchymal stem cells, they found that both proliferation and differentiation were promoted [48]. Our study was conducted on the murine pre-osteoblastic cell line, MC3T3-E1, using 50 Hz PEMF with 0.6 mT intensity and 30 min exposure duration. These parameters were based on our previous study where this set of specific parameters enhanced both proliferation and osteogenic gene expression of MC3T3-E1 cells cultured on tissue culture polystyrene (TCPS) [22]. Similar effects were also observed in our current study. PEMF exposure alone significantly enhanced cell proliferation, an effect that was not negated by the PVDF coating. It was suggested by Sun et al. that PEMF treatment could alter cell cycle progression, resulting in the early start of the DNA synthesis phase and increased cell proliferation [21]. Furthermore, Deng et al. demonstrated that PEMF alters progression from G_1_ to S phase by changing voltage-gated delayed rectifier K^+^ current, the Ca^2+^ activated K^+^ channels, and the cell cycle-dependent expression of ion channels [49]. PEMF also promoted the expression of all osteogenic genes tested in our study. *RUNX2* plays a crucial role in both intramembranous and endochondral ossification and serves as an activator of *BSP*, *OC*, and *OP* transcription [50]. The upregulation of *RUNX2* gene expression showed the ability of PEMF to activate bone regeneration. *BSP* is highly expressed during early mineralization, while *OP* and *OC* are highly expressed in immature and mature osteoblast, respectively, and upregulated during the late stage of osteogenesis [51,52]. *BSP* upregulation by PEMF or PVDF was observed on day 28, suggesting both PEMF and PVDF could promote the onset of early osteogenic differentiation. A synergistic effect of PEMF and PVDF to stimulate late osteogenic differentiation was also shown in our study by the upregulation of *OC* and *OP* gene expression. In a previous study, Kaivosoja et al. also reported that PEMF exposure (15 Hz, 1 Gauss EM field, 24 h/day) promoted the expression of *RUNX2*, *OC*, and *OP* by less than twofold [53]. Additionally, Mirzaei et al. demonstrated an enhancement in the expression levels of *RUNX2*, *OC*, and *OP* when dental pulp stem cells cultured on poled PVDF nanofibrous films were exposed to PEMF (50 Hz, 1 mT, 6 h/day) [54]. The upregulation of osteogenic differentiation gene markers by PEMF was reported to be associated with the triggered cascades of signaling pathways (Figure 7b), such as Ca^2+^ signaling, the Wnt/β-catenin signaling pathway, and the MAPK pathway [55]. Among different possible mechanisms, intracellular Ca^2+^ messaging is one of the main modes to translate PEMF signals into biological responses. PEMF depolarizes cells by activating voltage-gated Ca^2+^ channels and forced movement of calcium ions across both the cell membrane and endoplasmic reticulum, resulting in increased cytosolic calcium. Subsequently, membrane depolarization is able to alter various calcium-related signaling pathways such as Ca^2+^ and Ca^2+^-calmodulin (CaM)-dependent protein kinase [56,57,58,59]. To this effect, both electric fields and magnetic fields contribute. A study by Funk et al. revealed that electric fields exert forces at the surface of cell membranes, whereas magnetic fields penetrate deeper into the cells to achieve this outcome [60].

## 4. Materials and Methods

### 4.1. Scaffold Fabrication

PCL-TCP films of 0.2 mm thickness were prepared by heat-pressing medical-grade PCL-TCP (Osteopore International, Singapore) between two heated metal plates at 60 °C and 1 ton for 3 min, followed by cooling to room temperature via convection cooling by switching off the heaters. Circular samples with a diameter of 12.5 mm were punched out. PVDF homopolymer (Solvay, Brussels, Belgium) was dissolved in DMSO (Sigma-Aldrich, St. Louis, MO, USA) using heat at temperatures in the range from 50 to 220 °C for 15 min and cooled to room temperature. The final solution concentration was 50 mg/mL. Subsequently, the PCL-TCP scaffolds were dip-coated using the PVDF/DMSO solutions. This allows PVDF films of a few micrometers to be formed on PCL-TCP scaffolds. The scaffolds were then conditioned inside a closed chamber (0.75 L) with a relative humidity of 10%, 16%, and 56% at room temperature, prior to being kept at −20 °C for 2 h. The humidity changes throughout the 2 h were displayed in Figure S1. The coated scaffolds were then dried at an ambient temperature of 23 °C and relative humidity of 56%.

For cell culture, the circular samples were sticked to cover glass (diameter = 13 mm, Paul Marienfeld, Lauda- Königshofen, Germany) using bioinert silicone glue (Bostik, Colombes, France) and air dried. The scaffolds were sterilized by immersion in ethanol for 1.5 h and exposure to ultraviolet light (UV) (253.7 nm, 30 W, Sankyo Denki, Kanagawa, Japan) for 45 min on each side. The scaffolds were then rinsed in phosphate buffer saline (PBS) (Sigma-Aldrich) three times and alpha minimum essential medium (α-MEM) (Gibco; Life Technologies, Carlsbad, CA, USA) once to remove any ethanol residue prior to cell seeding.

### 4.2. Piezoelectric Characterization

Phases of PVDF film were characterized by XRD (Bruker D8 Advance, Billerica, MA, USA) using Cu Kα radiation operated at 40 kV and 40 mA. The continuous scan was conducted from 10° to 60°. The fraction of β phase existing in the crystalline phase was obtained using attenuated total reflectance fourier-transformed infrared (ATR-FTIR) absorption bands at 766 and 840 cm^−1^, which are characteristic of α and β phases, respectively, as shown in Equation (1) [61].

(1) $$F\left(\beta \right)=\frac{X_{\beta }}{X_{\alpha }+X_{\beta }}=\frac{A_{\beta }}{(K_{\beta }+K_{\alpha })A_{\alpha }+A_{\beta }}=\frac{A_{\beta }}{(1.26)A_{\alpha }+A_{\beta }}$$

where the absorption coefficient of α and β phases *K*_α_ = 6.1 × 10^4^ cm^2^/mol and *K*_β_ =7.7 × 10^4^ cm^2^/mol, *A*_α_ and *A*_β_ are absorbances at 766 and 840 cm^−1^, and *X*_α_ and *X*_β_ are the degree of crystallinity of each phase.

The degree of crystallinity (*X_c_*) was determined using DSC as the ratio of crystallization enthalpy between the sample and total crystalline PVDF, as shown in Equation (2) [62].

(2) $$X_{c}=\frac{\Delta H_{m}}{\Delta H_{o}}$$

where Δ*H_m_* is the crystallization enthalpy of the sample and Δ*H_o_* is the crystallization enthalpy of 100% crystalized PVDF Δ*H_o_* =104.50 J/g.

The absolute amount of piezoelectric β phase (%*β*) was estimated by Equation (3) [38]:

(3) $$\%\beta =F\left(\beta \right)\times X_{c}$$

The macroscopic piezoelectricity of the films and scaffolds was measured by stress piezoelectric coefficient *d*_33_. A *d*_33_ meter (APC International, Mill Hall, PA, USA) was used at an operating frequency of 110 Hz and a resolution of 0.1 pC/N. The coated samples were measured in the orthogonal direction to the film plane.

### 4.3. Ferroelectric Characterization

The ferroelectric behavior of the coated films was measured by macroscopic ferroelectric polarization–electric field (P–E) hysteresis measurement using a Sawyer–Tower circuit (Radiant Technologies, Albuquerque, NM, USA) on PVDF-coated PCL-TCP films. The samples were prepared by spin-coating PCL-TCP on indium tin oxide (ITO) coated glass slides and dip-coating 50 mg/mL PVDF/DMSO on PCL-TCP/ITO prior to conditioning the sample inside the closed chamber at three relative humidity (RH) levels at a temperature of −20 °C for 2 h, followed by air-drying. Finally, gold electrodes were sputter-coated on top of PVDF/PCL-TCP/ITO glass as the top electrode.

### 4.4. Surface and Bulk Film Characterization

Material and cell morphologies were studied by FESEM (JEOL JSM-6340F & 7600F, Tokyo, Japan). After cell culture, the films were removed from the culture medium, washed with PBS three times, and fixed with 3% glutaraldehyde for 1 h, following by dehydration in a series of ethanol solutions in water (50%, 70%, 90%, and 100%) and air drying overnight. All samples were mounted onto an SEM stub using carbon tape and sputter-coated using a gold target at a current of 20 mA for 30 s. The accelerating voltage was set at 5 kV. The surface topography and surface roughness were studied by AFM (Asylum Research AFM Cypher S, Santa Barbara, CA, USA) in non-contact tapping mode. All samples were prepared by mounting onto a stainless-steel disk and measured. Soft tip (NanoWorld, Neuchâtel, Switzerland) with an 8 ± 2 nm radius, 160 Hz resonance frequency, and 7.4 N/m force constant was used at a frequency of 1 Hz and area of 5 μm × 5 μm to generate the topography and roughness. The hydrophilicity of samples was studied by optical WCA measurement system (Dataphysics Instruments OCA 15Pro, Filderstadt, Germany). Here, 6 μL water drop was used for each measurement and the contact angles of each sample were recorded within 15 s after placing droplets. Accelerated degradation test of scaffolds coated at RH 56% over the period of 28 days was conducted using 3M NaOH in PBS solution. The results are displayed in Figure S3.

### 4.5. Cell Culture

Murine MC3T3-E1 Subclone 4 cells (CRL-2593) were (ATCC, Manassas, Virginia) supplied at passage 3 and used at passage 7 to 9. The MC3T3-E1 cells were expanded and cultured in supplier-recommended modified α-MEM supplemented with 10% fetal bovine serum (Gibco; Life Technologies), 100 U/mL penicillin, and 0.1 mg/mL streptomycin antibiotics (PenStrep; Life Technologies) in an incubator with 95% humidified air with 5% CO_2_ incubator. The osteogenic media contained a growth medium supplemented with 10 mM β-glycerophosphate, 0.2 mM ascorbic acid, and 10 nM dexamethasone (Sigma-Aldrich) [22]. The culture media was changed every 2–3 days. The cells were seeded at a seeding density of 1000 cells/cm^2^. After cell seeding, cells were allowed to adhere for 24 h before PEMF treatment started. PEMF-treated groups were exposed to PEMF for 30 min per day.

### 4.6. PEMF Instrumentation

**Figure 8:**
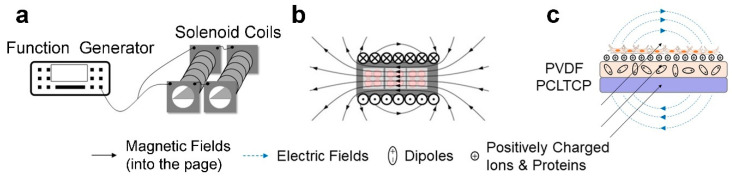
Experimental set-up. (**a**) Equipment set-up. (**b**) Arrangement of cell culture plates in solenoid coils. (**c**) Electric and magnetic fields’ orientation.

PEMF was induced in solenoid coils (inner diameter 7 cm, outer diameter 9 cm, length 32.5 cm, wire thickness 1 mm) powered by a function generator (Siglent SDG2042X, Shenzhen, China) with an average magnetic field intensity equal to 0.60 ± 0.01 mT, signal frequency of 50.00 ± 0.01 Hz, and pulse duration of 3.00 ms, as described in our previous paper [22]. The resistance of solenoid coils was at 16 Ω each and connected in parallel, which resulted in a total resistance of 8 Ω. The magnetic fields changing with time is shown in Figure S4 and the experimental setup is shown in Figure 8.

### 4.7. Cell Metabolic Activity

Metabolic activities of cells in growth media were evaluated by an alamarBlue colorimetric assay (Invitrogen, Thermo Fisher Scientific, Waltham, MA, USA) on day 3, 7, and 28 according to the manufacturer’s instructions. The cells were washed with phosphate-buffered saline (PBS) prior to immersion in 10% alamarBlue solution in fetal bovine serum (FBS)-free growth medium for 3 h in an incubator. The reduced solutions were transferred to a clear 96-well plate to be read in a microplate reader (SpectraMax, Molecular Devices, San Jose, CA, USA) at an excitation wavelength of 580 nm and emission wavelength of 600 nm.

### 4.8. Cell Proliferation

Cell proliferation in growth media was characterized by DNA quantification at different time points during the study period, measured by a Quant-iT PicoGreen dsDNA Assay Kit (Invitrogen; Thermo Fisher Scientific) according to the manufacturer’s protocol. In brief, cells were harvested at day 3, 7, and 28 following a 3 h enzymatic treatment using 0.1 w/v% collagenase and 0.1 v/v% trypsin, followed by three freeze–thaw cycles (lysing process). The harvested solution containing DNA was transferred to a black 96-well plate and mixed with quantification reagent from the assay kit. The fluorescence intensities of the sample solutions were read and compared with the fluorescence intensities of known DNA concentrations to yield the amount of DNA in the samples.

### 4.9. ALP Activity

The osteoblastic differentiation of MC3T3-E1 cells was assessed by measuring ALP activity in osteogenic media on day 7, 21, and 28 using a pNPP alkaline phosphatase assay kit (SensoLyte, Anaspec, Fremont, CA, USA). The solution that was enzymatically harvested as described in the cell proliferation section was used in ALP activity measurement as well. Here, 0.05 mL harvested solution and 0.05 mL pNPP solution were incubated together for 20 min at 37 °C. The absorbance of the mixed solutions was measured at 405 nm and normalized against absorbance value of day 7 PCL-TCP sample group to yield the relative ALP activity between sample groups.

### 4.10. OsteoImage

Mineralization of MC3T3-E1 cells was assessed by the OsteoImage kit (Lonza, Basel, Switzerland) based on fluorescent staining of hydroxyapatite in cell deposits. Briefly, cells cultured at different time points were fixed, stained with the fluorescent reagent, and measured at excitation wavelength 492 nm and emitted wavelength 520 nm and observed using a fluorescence microscope (Olympus, Shinjuku City, Tokyo, Japan).

### 4.11. Real-Time Polymerase Chain Reaction

The expression of osteogenic differentiation-related gene markers was evaluated by real-time polymerase chain reaction analysis (qRT-PCR) in cells growing in osteogenic media. On day 7 and 28, RNA was extracted using a RNeasy Protect Mini Kit (Qiagen, Hilden, Germany) and reversely transcribed to cDNA using an iScript cDNA Synthesis Kit (Bio-Rad, Hercules, CA, USA) with thermal cycler T100 (Bio-Rad). The qRT-PCR was performed on a CFX Connect Real-Time PCR system (Bio-Rad) with SsoAdvanced Universal SYBR Green supermix (Bio-Rad) as in our previous reports [22]. Five gene primers (AIT Biotech, Singapore) were used in the experiment, including glyceraldehyde-3-phosphate dehydrogenase (*GAPDH*), *RUNX2*, *BSP*, *OP*, and *OC* (listed in Table 2). The results presented were normalized against *GAPDH* as the endogenous housekeeping gene. The gene expression was calculated using the 2^−ΔΔCT^ method relative to the undifferentiated MC3T3-E1 controls (cDNA from undifferentiated MC3T3-E1 cells obtained from the same batch).

### 4.12. Statistical Analysis

All data are presented as mean ± standard deviation (SD) and differences were compared using the two-tailed Student’s *t*-test at a significance level of *p* < 0.05. Error bars represent the standard deviation (SD) of measurements within each sample. Triplicates are used in this study.

## 5. Conclusions

Piezoelectric PCL-TCP films with *d*_33_ = −1.2 pC/N were developed by incorporating PVDF as conformal piezoelectric coatings using a modified breath-figure method. The self-poling approach developed in this study represents a versatile solution for PVDF polarization at a low temperature when coated on material with low melting temperatures or complex structures such as macroporous three-dimensional scaffolds. In order to explore the possibility to synergistically stimulate bone-forming cells by mimicking the in vivo piezoelectric environment and exposing cells to external mechanical/electrical stimuli, piezoelectric scaffolds loaded with MC3T3-E1 cells were investigated under PEMF stimulation. The results suggested both PVDF and PEMF triggered osteoinduction and the combination of both stimulations enhanced the osteogenic differentiation potential of MC3T3-E1 cells, as indicated by enhanced cell proliferation and mineralization, as well as increased expression levels of *RUNX2*, *BSP*, *OC,* and *OP*. The combination of PVDF-coated PCL-TCP scaffolds and PEMF (50 Hz, 0.6 mT, 30 min/day) provides a powerful strategy for electrically augmented osteoinduction to be investigated for clinical application.

## Figures and Tables

**Table 1:** Summary of the effect of PEMF and PVDF coating on osteogenesis of MC3T3-E1 cells as compared with the control group of PCL-TCP scaffolds.

		PCLTCP + PEMF	PCLTCP + PVDF	PCLTCP + PEMF + PVDF
Cell Metabolic Activity		+	0	+
Cell Proliferation		++	0	++
Alkaline Phosphatase Activity		0	0	0
Cell Mineralization		0	+	+
Osteogenic Gene expression	*RUNX2*	+	0	+
	*BSP*	+	+	+
	*OC*	++	++	+++
	*OP*	+	0	++

Table legend: 0 = no significant effect; + = greatest effect smaller than twofold enhancement; ++ = greatest effect is between two- to fivefold enhancement; +++ = greatest effect greater than fivefold enhancement.

**Table 2:** The sequence of primers used in the study [63,64].

	Forward	Reverse
*GAPDH*	CGTCCCGTAGACAAAATGGT	AATGGCAGCCCTGGTGAC
*BSP*	TTTATCCTCCTCTGAAACGGT	GTTTGAAGTCTCCTCTTCCTCC
*RUNX2*	GCTATTAAAGTGACAGTGGACGG	GGCGATCAGAGAACAAACTAGG
*OP*	GATGAACAGTATCCTGATGCC	TTGGAATGCTCAAGTCTGTG
*OC*	CCGGGAGCAGTGTGAGCTTA	TAGATGCGTTTGTAGGCGGTC

## Data Availability

The data presented in this study are available on request from the corresponding author due to privacy.

## Supplementary Materials

The following are available online at https://www.mdpi.com/article/10.3390/ijms22126438/s1.

## References

[1] (2020). Bone Grafts and Substitutes Market—Global Industry Analysis, Size, Share, Growth, Trends, and Forecast 2020–2030.

[2] Hutmacher D.W., Schantz T., Zein I., Ng K.W., Teoh S.H., Tan K.C. (2001). Mechanical properties and cell cultural response of polycaprolactone scaffolds designed and fabricated via fused deposition modeling. J. Biomed. Mater. Res..

[3] Woodruff M.A., Hutmacher D.W. (2010). The return of a forgotten polymer—Polycaprolactone in the 21st century. Prog. Polym. Sci..

[4] Teoh S.H., Goh B.T., Lim J. (2019). Three-Dimensional Printed Polycaprolactone Scaffolds for Bone Regeneration Success and Future Perspective. Tissue Eng. Part A.

[5] Liu Y., Lim J., Teoh S.-H. (2013). Review: Development of clinically relevant scaffolds for vascularised bone tissue engineering. Biotechnol. Adv..

[6] Zhang Z.-Y., Teoh S.H., Chong W.-S., Foo T.-T., Chng Y.-C., Choolani M., Chan J. (2009). A biaxial rotating bioreactor for the culture of fetal mesenchymal stem cells for bone tissue engineering. Biomaterials.

[7] Ravichandran A., Lim J., Chong M.S.K., Wen F., Liu Y., Pillay Y.T., Chan J.K.Y., Teoh S.-H. (2016). In vitro cyclic compressive loads potentiate early osteogenic events in engineered bone tissue. J. Biomed. Mater. Res. Part B Appl. Biomater..

[8] Roseti L., Parisi V., Petretta M., Cavallo C., Desando G., Bartolotti I., Grigolo B. (2017). Scaffolds for Bone Tissue Engineering: State of the art and new perspectives. Mater. Sci. Eng. C.

[9] Remes A., Williams D. (1991). Relationship between chemotaxis and complement activation by ceramic biomaterials. Biomaterials.

[10] Nakielski P., Pawłowska S., Rinoldi C., Ziai Y., De Sio L., Urbanek O., Zembrzycki K., Pruchniewski M., Lanzi M., Salatelli E. (2020). Multifunctional Platform Based on Electrospun Nanofibers and Plasmonic Hydrogel: A Smart Nanostructured Pillow for Near-Infrared Light-Driven Biomedical Applications. ACS Appl. Mater. Interfaces.

[11] Zhang S., Chen Y., Huang Y., Dai H., Lin Y. (2020). Design and application of proximity hybridization-based multiple stimuli-responsive immunosensing platform for ovarian cancer biomarker detection. Biosens. Bioelectron..

[12] Municoy S., Echazú Álvarez M.I., Antezana P.E., Galdopórpora J.M., Olivetti C., Mebert A.M., Foglia M.L., Tuttolomondo M.V., Alvarez G.S., Hardy J.G. (2020). Stimuli-Responsive Materials for Tissue Engineering and Drug Delivery. Int. J. Mol. Sci..

[13] Fukada E., Yasuda I. (1957). On the Piezoelectric Effect of Bone. J. Phys. Soc. Jpn..

[14] Tandon B., Blaker J.J., Cartmell S.H. (2018). Piezoelectric materials as stimulatory biomedical materials and scaffolds for bone repair. Acta Biomater..

[15] Low Y.K.A., Zou X., Fang Y.M., Wang J.L., Lin W.S., Boey F.Y.C., Ng K.W. (2014). beta-Phase poly(vinylidene fluoride) films encouraged more homogeneous cell distribution and more significant deposition of fibronectin towards the cell-material interface compared to alpha-phase poly(vinylidene fluoride) films. Mater. Sci. Eng. C Mater. Biol. Appl..

[16] Ribeiro C.M.O., Pärssinen J., Sencadas V., Correia V.M.G., Miettinen S., Hytönen V.P., Lanceros-Méndez S. (2015). Dynamic piezoelectric stimulation enhances osteogenic differentiation of human adipose stem cells. J. Biomed. Mater. Res. Part A.

[17] Low Y.K.A., Meenubharathi N., Niphadkar N.D., Boey F.Y.C., Ng K.W. (2011). α- and β-Poly(Vinylidene Fluoride) Evoke Different Cellular Behaviours. J. Biomater. Sci. Polym. Ed..

[18] Szewczyk P.K., Gradys A., Kim S.K., Persano L., Marzec M., Kryshtal A., Busolo T., Toncelli A., Pisignano D., Bernasik A. (2020). Enhanced Piezoelectricity of Electrospun Polyvinylidene Fluoride Fibers for Energy Harvesting. ACS Appl. Mater. Interfaces.

[19] Ribeiro C., Correia V., Martins P., Gama F., Lanceros-Mendez S. (2016). Proving the suitability of magnetoelectric stimuli for tissue engineering applications. Colloids Surfaces B Biointerfaces.

[20] Markov M. (2015). Electromagnetic Fields in Biology and Medicine.

[21] Sun L.-Y., Hsieh D.-K., Yu T.-C., Chiu H.-T., Lu S.-F., Luo G.-H., Kuo T.K., Lee O.K., Chiou T.-W. (2009). Effect of pulsed electromagnetic field on the proliferation and differentiation potential of human bone marrow mesenchymal stem cells. Bioelectromagnetics.

[22] Suryani L., Too J.H., Hassanbhai A.M., Wen F., Lin D.J., Yu N., Teoh S.H. (2019). Effects of Electromagnetic Field on Proliferation, Differentiation, and Mineralization of MC3T3 Cells. Tissue Eng. Part C Methods.

[23] Galli C., Pedrazzi G., Mattioli-Belmonte M., Guizzardi S. (2018). The Use of Pulsed Electromagnetic Fields to Promote Bone Responses to Biomaterials In Vitro and In Vivo. Int. J. Biomater..

[24] Khare D., Basu B., Dubey A.K. (2020). Electrical stimulation and piezoelectric biomaterials for bone tissue engineering applications. Biomaterials.

[25] Martins P., Lopes A., Lanceros-Mendez S., Martins P., Lopes A., Lanceros-Mendez S. (2014). Electroactive phases of poly(vinylidene fluoride): Determination, processing and applications. Prog. Polym. Sci..

[26] Ruan L., Yao X., Chang Y., Zhou L., Qin G., Zhang X. (2018). Properties and Applications of the beta Phase Poly(vinylidene fluoride). Polymers.

[27] Wang H., Liu Z., Wang E., Yuan R., Gao D., Zhang X., Zhu Y. (2015). A robust superhydrophobic PVDF composite coating with wear/corrosion-resistance properties. Appl. Surf. Sci..

[28] Martins P., Ribeiro S., Sencadas V., Gomes A.C., Gama F.M., Lanceros-Méndez S. (2013). Effect of poling state and morphology of piezoelectric poly(vinylidene fluoride) membranes for skeletal muscle tissue engineering. RSC Adv..

[29] He X., Yao K. (2006). Crystallization mechanism and piezoelectric properties of solution-derived ferroelectric poly(vinylidene fluoride) thin films. Appl. Phys. Lett..

[30] Chen S., Yao K., Tay F.E.H., Chew L.L.S. (2010). Comparative investigation of the structure and properties of ferroelectric poly(vinylidene fluoride) and poly(vinylidene fluoride-trifluoroethylene) thin films crystallized on substrates. J. Appl. Polym. Sci..

[31] Kang S.J., Park Y.J., Sung J., Jo P.S., Park C., Kim K.J., Cho B.O. (2008). Spin cast ferroelectric beta poly(vinylidene fluoride) thin films via rapid thermal annealing. Appl. Phys. Lett..

[32] Martín J., Zhao D., Lenz T., de Leeuw D.M., Stingelin N. (2017). Solid-state-processing of δ-PVDF. Mater. Horiz..

[33] Parida K., Bhavanasi V., Kumar V., Bendi R., Lee P.S. (2017). Self-powered pressure sensor for ultra-wide range pressure detection. Nano Res..

[34] Lee H., Hwang H., Kim Y., Jeon H., Kim G. (2014). Physical and bioactive properties of multi-layered PCL/silica composite scaffolds for bone tissue regeneration. Chem. Eng. J..

[35] Li L.-J., Kim S.-N., Cho S.-A. (2016). Comparison of alkaline phosphatase activity of MC3T3-E1 cells cultured on different Ti surfaces: Modified sandblasted with large grit and acid-etched (MSLA), laser-treated, and laser and acid-treated Ti surfaces. J. Adv. Prosthodont..

[36] Heremans P., Gelinck G.H., Müller R., Baeg K.-J., Kim D.-Y., Noh Y.-Y. (2011). Polymer and Organic Nonvolatile Memory Devices†. Chem. Mater..

[37] Benz M., Euler W.B., Gregory O.J. (2001). The Influence of Preparation Conditions on the Surface Morphology of Poly(vinylidene fluoride) Films. Langmuir.

[38] Low Y.K.A., Tan L.Y., Tan L.P., Boey F.Y.C., Ng K.W. (2013). Increasing solvent polarity and addition of salts promote β-phase poly(vinylidene fluoride) formation. J. Appl. Polym. Sci..

[39] Chen S., Li X., Yao K., Tay F.E.H., Kumar A., Zeng K. (2012). Self-polarized ferroelectric PVDF homopolymer ultra-thin films derived from Langmuir–Blodgett deposition. Polymers.

[40] Garain S., Sinha T.K., Adhikary P., Henkel K., Sen S., Ram S., Sinha C., Schmeißer D., Mandal D. (2015). Self-Poled Transparent and Flexible UV Light-Emitting Cerium Complex–PVDF Composite: A High-Performance Nanogenerator. ACS Appl. Mater. Interfaces.

[41] Karan S.K., Bera R., Paria S., Das A.K., Maiti S., Maitra A., Khatua B.B. (2016). An Approach to Design Highly Durable Piezoelectric Nanogenerator Based on Self-Poled PVDF/AlO-rGO Flexible Nanocomposite with High Power Density and Energy Conversion Efficiency. Adv. Energy Mater..

[42] Weber N., Lee Y.-S., Shanmugasundaram S., Jaffe M., Arinzeh T. (2010). Characterization and in vitro cytocompatibility of piezoelectric electrospun scaffolds. Acta Biomater..

[43] Zhou Z., Li W., Zhengnan Z., Qian L., Tan G., Ning C. (2016). Polarization of an electroactive functional film on titanium for inducing osteogenic differentiation. Sci. Rep..

[44] Park J.B., Lakes R.S. (1979). Biomaterials: An Introduction.

[45] Uzieliene I., Bernotas P., Mobasheri A., Bernotiene E. (2018). The Role of Physical Stimuli on Calcium Channels in Chondrogenic Differentiation of Mesenchymal Stem Cells. Int. J. Mol. Sci..

[46] Pärssinen J., Hammarén H., Rahikainen R., Sencadas V.J.G.S., Ribeiro C., Vanhatupa S., Miettinen S., Lanceros-Méndez S., Hytönen V.P. (2015). Enhancement of adhesion and promotion of osteogenic differentiation of human adipose stem cells by poled electroactive poly(vinylidene fluoride). J. Biomed. Mater. Res. Part A.

[47] Jansen J.H., van der Jagt O.P., Punt B.J., Verhaar J.A., van Leeuwen J.P., Weinans H., Jahr H. (2010). Stimulation of osteogenic differentiation in human osteoprogenitor cells by pulsed electromagnetic fields: An in vitro study. BMC Musculoskelet. Disord..

[48] Ferroni L., Gardin C., Dolkart O., Salai M., Barak S., Piattelli A., Amir-Barak H., Zavan B. (2018). Pulsed electromagnetic fields increase osteogenetic commitment of MSCs via the mTOR pathway in TNF-α mediated inflammatory conditions: An in-vitro study. Sci. Rep..

[49] Deng X.L., Lau C.P., Lai K., Cheung K.F., Lau G.K., Li G.R. (2007). Cell cycle-dependent expression of potassium channels and cell proliferation in rat mesenchymal stem cells from bone marrow. Cell Prolif..

[50] Bruderer M., Richards R.G., Alini M., Stoddart M.J. (2014). Role and regulation of RUNX2 in osteogenesis. Eur Cell Mater.

[51] Atkins G.J., Findlay D.M., Anderson P.H., Morris H.A., Feldman D., Pike J.W., Adams J.S. (2011). Target Genes: Bone Proteins. Vitamin D.

[52] Komori T. (2010). Regulation of Osteoblast Differentiation by Runx2. Osteoimmunology.

[53] Kaivosoja E., Sariola V., Chen Y., Konttinen Y.T. (2012). The effect of pulsed electromagnetic fields and dehydroepiandrosterone on viability and osteo-induction of human mesenchymal stem cells. J. Tissue Eng. Regen. Med..

[54] Mirzaei A., Saburi E., Enderami S.E., Barati Bagherabad M., Enderami S.E., Chokami M., Shapouri Moghadam A., Salarinia R., Ardeshirylajimi A., Mansouri V. (2019). Synergistic effects of polyaniline and pulsed electromagnetic field to stem cells osteogenic differentiation on polyvinylidene fluoride scaffold. Artif. Cells Nanomed. Biotechnol..

[55] Yuan J., Xin F., Jiang W. (2018). Underlying Signaling Pathways and Therapeutic Applications of Pulsed Electromagnetic Fields in Bone Repair. Cell. Physiol. Biochem..

[56] Titushkin I., Rao V., Cho M. (2004). Mode- and Cell-Type Dependent Calcium Responses Induced by Electrical Stimulus. IEEE Trans. Plasma Sci..

[57] Lacy-Hulbert A., Metcalfe J.C., Hesketh R. (1998). Biological responses to electromagnetic fields 1. FASEB J..

[58] Daish C., Blanchard R., Fox K., Pivonka P., Pirogova E. (2018). The Application of Pulsed Electromagnetic Fields (PEMFs) for Bone Fracture Repair: Past and Perspective Findings. Ann. Biomed. Eng..

[59] Sundelacruz S., Moody A.T., Levin M., Kaplan D.L. (2019). Membrane Potential Depolarization Alters Calcium Flux and Phosphate Signaling During Osteogenic Differentiation of Human Mesenchymal Stem Cells. Bioelectricity.

[60] Funk R.H.W., Monsees Tand Özkucur N. (2009). Electromagnetic effects—From cell biology to medicine. Prog. Histochem. Cytochem..

[61] Gregorio J.R., Cestari M. (1994). Effect of crystallization temperature on the crystalline phase content and morphology of poly(vinylidene fluoride). J. Polym. Sci. Part B Polym. Phys..

[62] Lanceros-Méndez S., Mano J.F., Costa A.M., Schmidt V.H. (2001). FTIR AND DSC STUDIES OF MECHANICALLY DEFORMED β-PVDF FILMS. J. Macromol. Sci. Part B.

[63] Yan X.-Z., Yang W., Yang F., Kersten-Niessen M., Jansen J.A., Both S.K. (2014). Effects of Continuous Passaging on Mineralization of MC3T3-E1 Cells with Improved Osteogenic Culture Protocol. Tissue Eng. Part C Methods.

[64] Cheng Y., Huang L., Wang Y., Huo Q., Shao Y., Bao H., Li Z., Liu Y., Li X. (2019). Strontium promotes osteogenic differentiation by activating autophagy via the the AMPK/mTOR signaling pathway in MC3T3-E1 cells. Int. J. Mol. Med..

