# Supplementary material for: Synergistic Effect of PVDF-Coated PCL-TCP Scaffolds and Pulsed Electromagnetic Field on Osteogenesis
Source: Int J Mol Sci. 2021 Jun 16;22(12):6438. doi: 10.3390/ijms22126438 (PMC8234164; doi:10.3390/ijms22126438)
Supplement: Supplementary file 1 [file ijms-22-06438-s001.zip › ijms-1222751-supplementary.pdf]

Supplementary Information for

Synergistic Effect of PVDF-Coated PCL-TCP Scaffolds and Pulsed Electromagnetic Field on Osteogenesis

1. Humidity Control in the closed chamber

The humidity level inside the closed chamber was monitored and recorded every minute throughout the conditioning period of 2 hours. Three measurements were taken for each relative humidity condition and the average of three measurements was plotted.

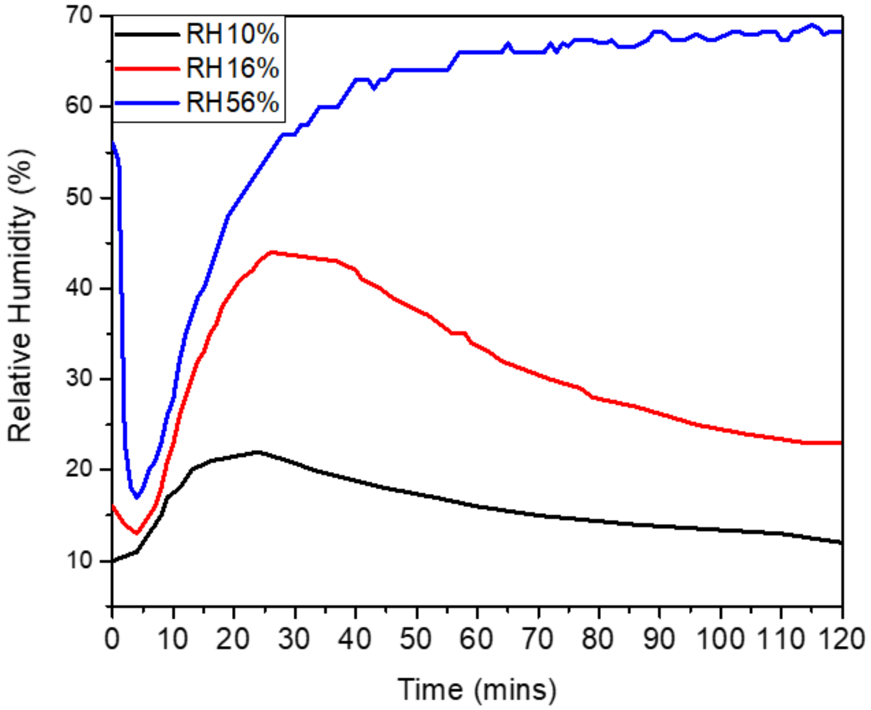

Supplementary Figure S1. Humidity change inside the closed chamber with different initial relative humidity (RH) conditions at 23 ℃ after conditioning at -20 ℃.

Relative humidity (RH) is expressed as the ratio of actual pressure of water vapor to the saturation vapor pressure at a given temperature. It is one of the most commonly used unit for humidity measurements. RH change inside the closed chamber was recorded in 2 hours as shown in supplementary Figure S1 and could be divided into

three phases. In the first phase, RH dropped rapidly to a lowest point for RH 16% and RH 56% groups, while for RH 10% group, RH increased slowly. This is possibly due to the existence of saturation vapor pressure for each given temperature. When vapor pressure exceeds the saturation vapor pressure (RH 100%), water vapor will start to condense [1]. It was reported when temperature reduced to below 0 ℃, the air in the closed container would be saturated and sweating and ice formation would occur [2]. This effect made the excess water vapor condensed thus reduced RH for RH16% and RH 56% groups with higher amount of water vapor. In the second phase, RH in all three conditions increased with time, which might be attributed to the decreased temperature. In a closed chamber with certain amount of vapor, when temperature decreases, the saturation vapor pressure drops, which leads to an increased relative humidity [2]. The highest RH reached for RH 10%, RH 16% and RH 56% are 22%, 44% and 69%. In the last phase, RH kept dynamic constant for RH56% while slowly dropped for RH10% and RH16%, which might be due to the crystallization of water vapor at the wall of the closed chamber. Overall, the samples will be exposed to three levels of RH in these three different conditions.

2. Surface Topography and Roughness of coated sample at various relative humidity

The representative AFM topography images and surface roughness are displayed in Supplementary Figure S2. With increasing humidity, more valleys and holes were observed in the topography, similar to that observed in FESEM images.

a

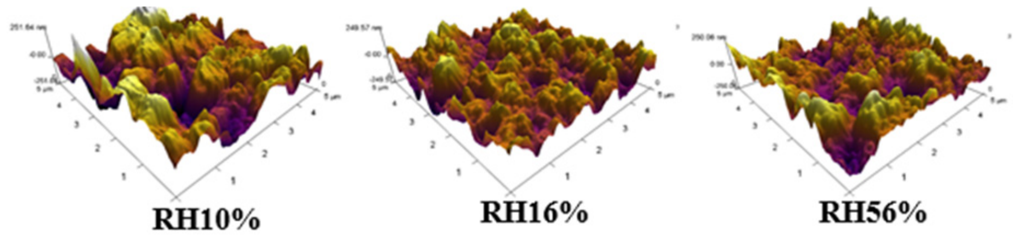

b

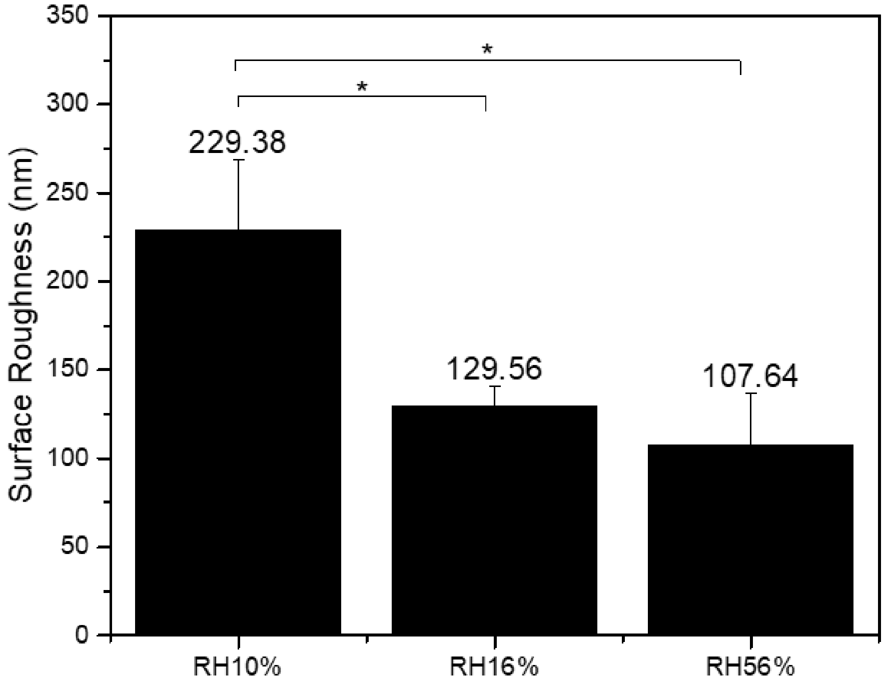

Supplementary Figure S2. (a) Topography and (b) surface roughness comparison of PVDF-coated PCLTCP fabricated at relative humidity of 10%, 16% and 56%. (*p<0.05, n=3)

However, the surface roughness exhibited a decreasing trend with increasing humidity. It was reported that the surface roughness depended upon solvent concentration, dissolution temperature and relative humidity during film formation. High dissolution temperature and high relative humidity during film formation resulted in rougher surface. In our experiments, as the dissolution temperature and relative humidity during film formation were same for all groups, the factor affecting surface roughness would be solvent concentration. It was reported when solvent concentration increased, larger amount of the solvent-PVDF complex were created, yielding rougher surface

[3]. In our experiments, with increasing humidity during freezing, more water molecules sank into the polymer chain and reduced the DMSO concentration, which might subsequently result in less DMSO-PVDF complex and a smoother surface.

3. Accelerated Degradation Test

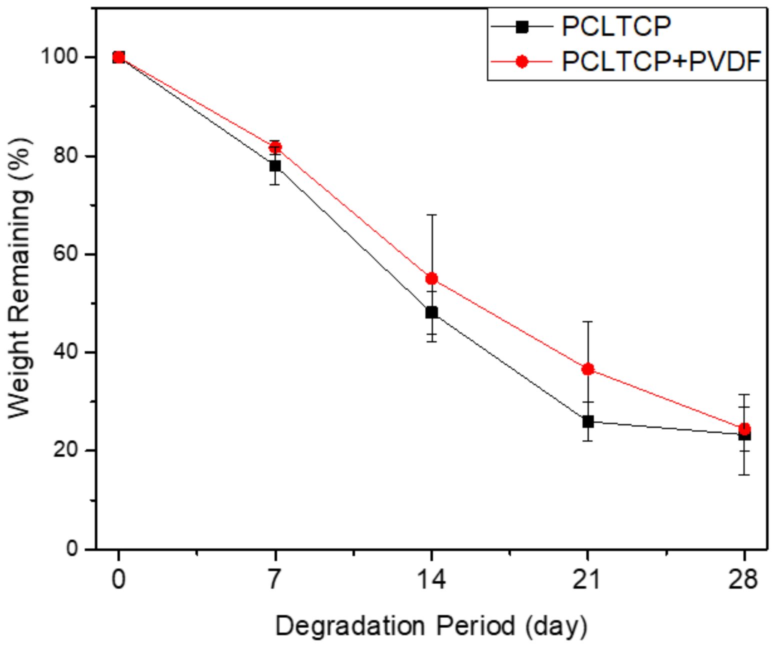

Supplementary Figure S3. Accelerated degradation test in 3M NaOH in PBS solution over the period of 28 days. (*$p<0.05$, n=3)

Accelerated degradation test was carried out to assess the rate of degradation. Scaffolds were prepared using a hole puncher to obtain circular films of 1.25 cm diameter. 1.5 mL aging solution of 3M NaOH in 1x phosphate-buffered saline (PBS) was pipetted into each Eppendorf tube with a scaffold and shook (Orbi-Shaker™ Jr.; Benchmark Scientific) for 2 hours daily at 100 rpm. At days 7, 14, 21, and 28, the scaffolds were washed with absolute Ethanol and left to dry before weighing. The results indicated the application of PVDF coating did not affect the degradation significantly though a slight slower trend was observed here.

4. Generation of β-PVDF

Supplementary Table S1

Diffraction crystal planes and angles of the PVDF.

Dissolving T.	Peak 2θ (°)	Crystal plane
PVDF powder	17.7, 18.3, 19.9, 26.5	(100), (020), (110), (021)
50 ℃	18.5, 20.2, 26.2	(020), (110/200), (021)
80 ℃	18.6, 20.2	(020), (110/200)
100 ℃	20.6	(110/200)
130 ℃	20.2	(110/200)
160 ℃	20.4	(110/200)
190 ℃	20.6	(110/200)
220 ℃	20.5	(110/200)

Supplementary Table S2.

The effect of dissolution temperature on the melting temperature (Tm), crystallization enthalpy (ΔHm), crystallinity (Xc), fraction of β phase (F(β)) in crystal region, and amount of β phase (%β) in overall semi-crystalline PVDF polymer.

Dissolution T.	Tm (℃)	ΔH_m (J/g)	X_c (%)	F(β) (%)	%β (%)
50 ℃	169.1	73.7	70.5	48.5	34.1
80 ℃	169.4	75.1	71.8	68.5	49.3
100 ℃	169.9	80.9	77.4	78.7	61.0
130 ℃	169.6	80.0	76.5	79.4	60.7
160 ℃	169.9	79.7	76.2	77.1	58.7

190 ℃	169.8	80.7	77.3	76.7	59.3
220 ℃	169.9	80.0	76.6	76.9	58.8

5. Self-polarization of PVDF

Supplementary Table S3.

Remnant polarization, Maximum polarization, and coercive field of PVDF-coated PCLTCP film under RH of 10%, 16% and 56% under the applied field up to 50kV/cm

Relative Humidity (%)	Pmax (μC/cm^2)	Pr (μC/cm^2)	Ec (kV/mm)
10	0.60	0.06	50
16	0.63	0.08	65
56	0.69	0.09	63

Supplementary Table S4.

Piezoelectric coefficient d_{33} and fraction of β phase of PVDF-coated PCLTCP film under RH of 10%, 16% and 56%

Relative Humidity (%)	d_{33} (pC/N)	S.D (pC/N)	Fraction of β phase (%)	S.D (%)
10	0.5	0.1	81.1	1.8
16	0.7	0.1	79.7	2.2
56	1.2	0.3	79.8	3.3

Supplementary Table S5.

Water Contact Angle Measurements of PVDF-coated PCLTCP film under RH of 10%, 16% and 56%

Relative Humidity (%)	Water Contact Angle (°)	Representative Water Droplet
10	119.9±4.4	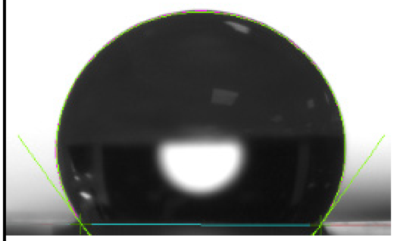
16	120.6±8.3	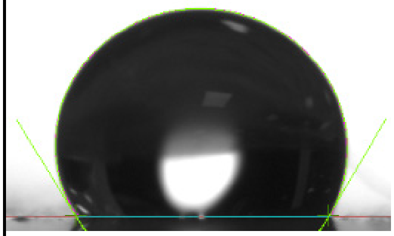
56	114.1±2.3	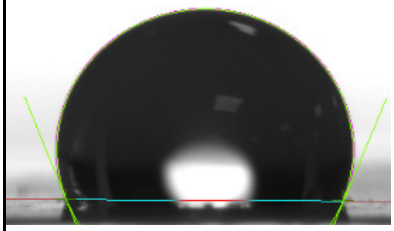

The wettability of PVDF coated PCLTCP films exhibited hydrophobic nature similar to what was reported in literature. [4, 5] There was no significant difference between water contact angles of three conditions. However, a slight decreasing trend presented which might be attributed to increased pore size and polarization of the film due to the increased surface charges in the more poled samples [6].

6. Calculation for induced mechanical strain by piezoelectric response of PVDF-coated PCL-TCP

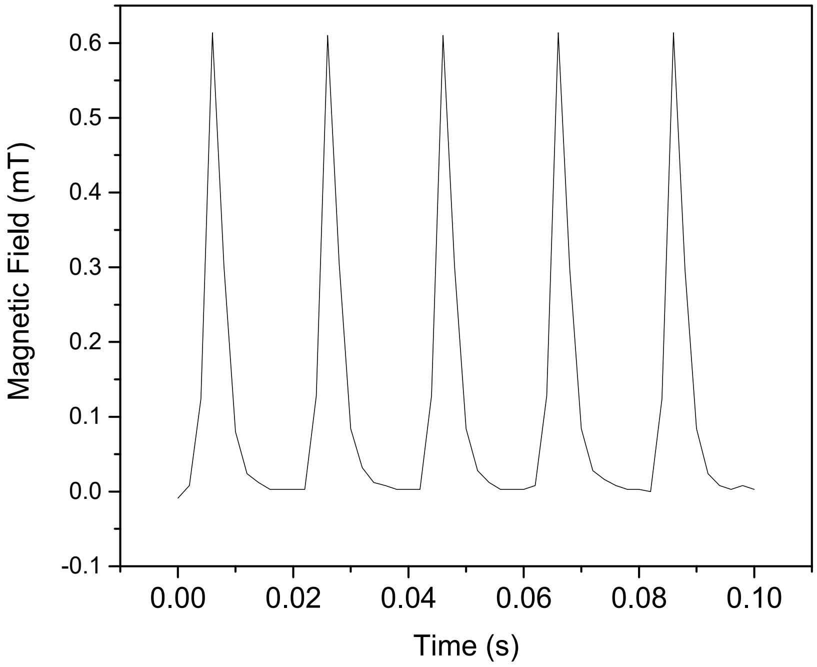

Supplementary Figure S4. Magnetic field changing with time.

The magnetic flux through a circular path with a radius of r is [7]:

$$m = BA = B\pi r^2 \quad \text{(S1)}$$

Where *B* is magnetic field, *A* is the circular area.

The induced field $\vec{E}$ is tangent to this path and constant on the path due to the cylindrical symmetry of the system, thus [7]:

$$\left| \oint \vec{E} d\vec{l} \right| = \left| \frac{dm}{dt} \right| \quad \text{(S2)}$$

$$E(2\pi r) = \frac{d}{dt}(B\pi r^2) \quad \text{(S3)}$$

$$E(2\pi r) = \pi r^2 \frac{dB}{dt} \quad \text{(S4)}$$

The induced field is calculated as:

$$E = \frac{\mathrm{r}}{2} \frac{dB}{dt} \quad \text{(S5)}$$

When the induced field was applied to PVDF-coated PCL-TCP film, the reverse piezoelectric effect could be formulated as [8]:

$$S_{33} = d_{33} \times E \qquad \text{(S6)}$$

where S_{33} is the mechanical strain produced, d_{33} is the piezoelectric coefficient and E is the induced electric field.

All samples (the middle point of the circular sample) were placed at a position of 0.016 m away from the solenoid coil center axis. Therefore, r equals to 0.016 m in our study. The derivative of magnetic fields versus time was taken to calculate induced electric field E using equation S5. The maximum magnitude of E of each pulse were averaged and substituted into equation S6-7 to calculate the peak strain induced. The magnitude of peak strain induced is estimated to be 6.4 to 7.3 x 10^{-10} ppm (μm/m).

Reference

1. Korotcenkov, G., Handbook of Humidity Measurement: Methods, Materials and Technologies, Vol. 1: Spectroscopic Methods of Humidity Measurement, CRC Press, Boca Raton, USA, 2018. **2018**.
2. Lassen, L., Influence of temperature on relative humidity within confined spaces with and without a desiccant. **1945**.
3. Benz, M., W.B. Euler, and O.J. Gregory, The Influence of Preparation Conditions on the Surface Morphology of Poly(vinylidene fluoride) Films. *Langmuir,* **2001**. 17(1): p. 239-243.
4. Wang, H., Z. Liu, et al., A robust superhydrophobic PVDF composite coating with wear/corrosion-resistance properties. *Applied Surface Science,* **2015**. 332: p. 518-524.
5. Wang, Z., H. Yu, et al., Novel GO-blended PVDF ultrafiltration membranes. *Desalination,* **2012**. 299: p. 50-54.
6. Martins, P.M., S. Ribeiro, et al., Effect of poling state and morphology of piezoelectric poly(vinylidene fluoride) membranes for skeletal muscle tissue engineering. *RSC Advances,* **2013**. 3(39): p. 17938-17944.
7. Ling, S.J., Induced Electric Fields, in *University Physics*. 2016.
8. Arnau, A. and D. Soares, Fundamentals of Piezoelectricity, in *Piezoelectric Transducers and Applications*, A.A. Vives, Editor. 2008, Springer Berlin Heidelberg: Berlin, Heidelberg. p. 1-38.
